# Supplementary material for: Genome sequence-based species classification of Enterobacter cloacae complex: a study among clinical isolates
Source: Microbiol Spectr. 2024 Apr 30;12(6):e04312-23. doi: 10.1128/spectrum.04312-23 (PMC11237491; doi:10.1128/spectrum.04312-23)
Supplement: Table S1 — Type strain genomes for ECC. [file spectrum.04312-23-s0001.docx]

Supplemental Material

**Table S1** Type strain genomes for ECC.

| **Type strain** | **Accession no.** |
| --- | --- |
| *E. asburiae* ATCC 35953 | CP011863 |
| *E. hormaechei* ATCC 49162 | QZCT00000000 |
| *E. hormaechei* subsp. *steigerwaltii* DSM 16691 | AEXB00000000 |
| *E. hormaechei* subsp. *oharae* DSM 16687 | JCKW00000000 |
| *E. hormaechei* subsp. *hormaechei* ATCC 49162 | QZCT00000000 |
| *E. hormaechei* subsp. *hoffmanii* DSM 14563 | CP001918 |
| *E. hormaechei* subsp. *xiangfangensis* LMG 27195 | AP019007 |
| *E. kobei* UCI 24 | POVL00000000 |
| *E. cloacae* ATCC 13047 | MKEQ00000000 |
| *E. bugandensis* EB-247 | QZCS00000000 |
| *E. cancerogenus* JY65 | SJON00000000 |
| *E. chengduensis* WCHECl-C4 | CP017184 |
| *E. chuandaensis* 090028 | CP017279 |
| *E. ludwigii* EN-119 | LXES00000000 |
| *E. quasihormaechei* WCHEQ120003 | SJOO00000000 |
| *E. roggenkampii* DSM 16690 | FYBI00000000 |
| *E. huaxiensis* 090008 | CP017186 |
| *E. mori* LMG 25706 | CP017179 |
| *E. oligotrophica* CCA6 | MKEQ00000000 |
| *E. sichuanensis* WCHECL1597 | CP017183 |
| *E. soli* ATCC BAA-2102 | CP017180 |
| *E. wuhouensis* WCHEs120002 | CP011863 |
